# Herpes simplex virus type 1 infection leads to neurodevelopmental disorder-associated neuropathological changes

**DOI:** 10.1371/journal.ppat.1008899

**Published:** 2020-10-22

**Authors:** Haowen Qiao, Moujian Guo, Jia Shang, Wen Zhao, Zhenyan Wang, Nian Liu, Bin Li, Ying Zhou, Ying Wu, Pu Chen

**Affiliations:** 1 Department of Biomedical Engineering, Wuhan University School of Basic Medical Sciences, Wuhan, Hubei, China; 2 Hubei Province Key Laboratory of Allergy and Immunology, Wuhan, Hubei, China; 3 State Key Laboratory of Virology, Wuhan University, Wuhan, Hubei, China; 4 Institute of Medical Virology, Wuhan University School of Basic Medical Sciences, Wuhan, Hubei, China; 5 Research Center for Medicine and Structural Biology of Wuhan University, Wuhan University, Wuhan, Hubei, China; University of Southern California, UNITED STATES

## Abstract

Neonatal herpes simplex virus type 1 (HSV-1) infections contribute to various neurodevelopmental disabilities and the subsequent long-term neurological sequelae into the adulthood. However, further understanding of fetal brain development and the potential neuropathological effects of the HSV-1 infection are hampered by the limitations of existing neurodevelopmental models due to the dramatic differences between humans and other mammalians. Here we generated in vitro neurodevelopmental disorder models including human induced pluripotent stem cell (hiPSC)-based monolayer neuronal differentiation, three-dimensional (3D) neuroepithelial bud, and 3D cerebral organoid to study fetal brain development and the potential neuropathological effects induced by the HSV-1 infections. Our results revealed that the HSV-1-infected neural stem cells (NSCs) exhibited impaired neural differentiation. HSV-1 infection led to dysregulated neurogenesis in the fetal neurodevelopment. The HSV-1-infected brain organoids modelled the pathological features of the neurodevelopmental disorders in the human fetal brain, including the impaired neuronal differentiation, and the dysregulated cortical layer and brain regionalization. Furthermore, the 3D cerebral organoid model showed that HSV-1 infection promoted the abnormal microglial activation, accompanied by the induction of inflammatory factors, such as TNF-α, IL-6, IL-10, and IL-4. Overall, our in vitro neurodevelopmental disorder models reconstituted the neuropathological features associated with HSV-1 infection in human fetal brain development, providing the causal relationships that link HSV biology with the neurodevelopmental disorder pathogen hypothesis.

## Author summary

HSV-1 is one of the most prevalent human pathogens that can spread into the fetal central nervous system by maternal-fetal transmission, and thus resulting in long-term neurological sequelae in adult, including cognitive dysfunction and learning disabilities. However, there is a very limited progress in understanding the role of HSV-1 on human fetal brain development due to limited access to fetal human brain tissue as well as the limitations of existing neurodevelopmental and infection models. Here, we generated the in vitro neurodevelopmental disorder models including hiPSC-based monolayer neuronal differentiation, three-dimensional (3D) neuroepithelial bud, and 3D cerebral organoid to study the neurodevelopmental disorder-associated neuropathological changes with HSV-1 infection in human fetal brain development. Our results revealed that HSV-1 infection led to impaired neural differentiation and dysregulated neurogenesis in the fetal neurodevelopment. Additionally, HSV-1 infection impaired neuronal differentiation and dysregulated brain regionalization in our cerebral organoid model. Furthermore, the cerebral organoid model showed that HSV-1 infection led to the abnormal microglial proliferation and activation, accompanied by the induction of inflammatory factors including TNF-α, IL-6, IL-10, and IL-4. Taken together, our study provides novel evidence that HSV-1 infection impaired human brain development and contributed to neurodevelopmental disorder pathogen hypothesis, and would have implications for raising the therapeutic opportunities for targeting of viral reservoirs relevant to neurodevelopmental disorder.

## Introduction

Neurodevelopmental disorders (NDDs)[1] are a group of conditions that interfere with the central nervous system (CNS) development at an early age. Developmental brain dysfunction causes later neuropsychiatric problems that persist into adulthood, including the attention-deficit/hyperactivity disorder (ADHD), autism spectrum disorder (ASD), learning disabilities, and intellectual disability[2]. Numerous epidemiological reports[3] suggest an association between NDDs and prenatal exposure to viral pathogens. The viral infection causes the brain changes, resulting in alterations in brain structure[4], impairments in synaptic function and transmission[5], immune alterations[6], and behavioral impairments[7] in the offspring. Though the pathogenesis of NDDs has not been completely understood, the increasing evidence support NDD pathogen hypothesis that the viral pathogens induce prolonged alterations in the offspring’s brain, which in turn may mediate the behavior and neurocognition.

Herpes simplex virus (HSV) is one of the most prevalent human pathogens worldwide[8]. HSV is categorized into 2 types: HSV-1 which is mainly transmitted by oral-to-oral contact to cause oral herpes and also cause genital herpes, and HSV-2 which is a sexually transmitted infection that causes genital herpes[9]. Both HSV-1 and HSV-2 are highly prevalent and capable of establishing lifelong infection[10]. Although HSV infections are generally confined to the oro-labial and genital skin and mucosa, the viruses are the most frequent cause of sporadic encephalitis, a potential deadly infection of the CNS[11]. Notably, HSV-1 is more likely than HSV-2 to be transmitted to the neonate[12]. HSV-1 infection is particularly common in adolescents and adults, and carries the risk of mother-to-child transmission when occurring in pregnant women[13]. Fetal HSV-1 infections can result in necrotizing encephalitis and even death in newborns[14]. Though the fetal HSV-1 infections occur less frequently, especially considering the high prevalence of HSV infection in the general population, up to 80% of positive herpetic condition is neglected by the pregnant woman[15]. The risk of maternal transmission of the virus to the fetus or neonate has become a major health concern due to the increased incidence of HSV infections in the pregnant women. Moreover, the accumulating studies[16] raise the possibility that the fetal HSV-1 infections during pregnancy are associated with risk of neurodevelopmental disorders, thus result in long-term neurological sequelae, including cognitive dysfunction, learning disabilities, and dementia in adult. However, the physiological features of the neurodevelopmental disorder associated with HSV-1 infections remain unclear.

Current understanding of the effect of HSV-1 infections on the human brain, especially fetal brain during pregnancy, is highly dependent on animal models which allow us to study different aspects of the lytic infection, latency, reactivation, and neuropathogenesis in vivo[17]. However, the human brain contains 100 billion neurons and 10 times more glial cells[18], which form an elaborate and determined pattern of neural circuits in adult individuals. This complexity is also reflected in the early stages of human fetal brain development. Specifically, the neurogenic outer radial glia (oRGs) which translocate to an additional outer subventricular zone (oSVZ) undergo mitosis to generate vastly more proliferative neuronal progenitors that, in turn, differentiate into more neurons[19]. Notably, oRGs only present to a limited degree in rodents, which lead to the interspecies differences in the striking expansion in neuronal output and brain size between humans and animal models[20,21]. Thus, the findings from rodent models failed to recapitulate some critical features of the developing human brain, and there have been limitations in extrapolating data from mouse model to HSV-1 pathogenesis in the human fetal brain. Given the dramatic differences between humans and other mammals, in terms of polarized neuroepithelium, neuron-glia interactions, intricate neurodevelopment, and spatiotemporal self-organization, it is difficult to study the human brain prenatal HSV-1 infection at various brain developmental stages.

Human physiologically and pathologically relevant in-vitro models will be vital to understand how HSV-1 interactions impact the human fetal CNS development. Paola Arlotta and his colleagues performed single-cell RNA sequencing analysis of brain organoids, finding that the individual brain organoids reproducibly form cell diversity of the human cerebral cortex[22]. Specifically, brain organoids generated cellular diversity of the human cerebral cortex following a precise and reproducible trajectory, such as oRGs. Each organoid distributed similarly along the pseudotemporal ordering of cell types approximated that of in vivo human brain development, indicating that their transcriptional profiles were similar to human fetal brain. Furthermore, this research calculated mutual information scores between cluster assignments and sample identity in the single-cell RNA-sequencing datasets including the brain organoids, human cortex, and mouse cortex, suggested that the degree of variation in the dorsally patterned brain organoid samples was similar to that of the human brain datasets, compared to the mouse brain samples. Overall, the hiPSC-derived 3D cerebral organoid opens up a door to probe human brain development, including neuronal distribution, cytoarchitectural organization, and functional status in the embryonic neocortex. For example, it recapitulates the specific changes of in vivo human fetal brain as a result of the perinatal teratogen exposure, such as ZIKV infection[23] and prenatal nicotine exposure[24], revealing their great potential to study the fetal brain development and neurodevelopmental disorder.

In this work, we generated in vitro neurodevelopmental disorder models to investigate the influence of HSV-1 infections on fetal brain development and potential neuropathology. We found that the HSV-1 infection led to impaired neural differentiation and dysregulated neurogenesis in the fetal neurodevelopment. Our results identified that the HSV-1-infected brain organoids modelled the pathological features of the neurodevelopmental disorders in the human fetal brain, including impaired neuronal differentiation and dysregulated cortical layer and brain regionalization. Furthermore, the 3D cerebral organoid model showed that HSV-1 infection promoted the abnormal microglial proliferation and activation, accompanied by the induction of inflammatory factors, such as TNF-α, IL-6, IL-10, and IL-4. Taken together, our data provided compelling evidence that HSV-1 infection impaired human brain development and contributed to NDD pathogen hypothesis.

## Materials and methods

### Generation of monolayer neuronal differentiation models

The hiPSCs were cultured in mTeSR medium (STEMCELL Technologies) with Rho-associated protein kinase (ROCK) inhibitor (STEMCELL Technologies). Neural differentiation was performed according to standard neurobiology protocols (Thermo Fisher Scientific). Specifically, the hiPSCs were cultured in mTeSR medium on day 1 of splitting with 15% confluency, then the cells were subsequently cultured in neural induction medium containing neurobasal medium and neural induction supplement (Life Technologies). On day 7 of neural induction, NSCs derived from hiPSCs were ready to be harvested and expanded.

For the neuron differentiation from NSCs, NSCs were seeded on 24-well plates coated with poly-ornithine and laminin at a density of 25,000 cells/cm^2^, in StemPro NSC SFM complete medium (Life Technologies) containing KnockOut DMEM/F-12 (Life Technologies), StemPro neural supplement (Life Technologies), recombinant human epidermal growth factor (EGF) (Life Technologies), basic fibroblast growth factor (bFGF) (Life Technologies), and GlutaMAX (Life Technologies). After 2 days of culturing in the NSC SFM medium, cells were differentiated in the neural differentiation medium containing neurobasal medium (Life Technologies), B-27 serum-free supplement (Life Technologies) and GlutaMAX. The neural differentiation medium was changed every 3**–**4 days for 21 days. The dibutyryl cAMP (Sigma) was added to the neural differentiation medium starting at day 7 of differentiation for 3 days.

### Generation of human cerebral organoids

The human cerebral organoids were generated from hiPSCs using a previously reported protocol[23]. Briefly, on day 0 of organoid culture, Accutase (Life Technologies) was used to dissociate the hiPSCs into single cells. A total of 2000 cells were then plated into hanging drop culture plates (InSphero AG) to form single embryoid body (EB) in the first 24 hours, then the EBs were transferred to ultra-low-attachment 96-well plates (Corning), in EB formation medium with low concentration bFGF (4 ng/ml) and ROCK inhibitor (50 mM). The EBs were fed every other day for 6 days then transferred to low adhesion 24-well plates (Corning) in induction media containing DMEM/F12 (Life Technologies), 1× N2 supplement (Life Technologies), 1% non-essential amino acids (Life Technologies), 2 mM GlutaMAX, and 1ug/ml heparin.

On day 11, the EBs were embedded in droplets of growth factor-reduced Matrigel (356231, Corning), and these droplets were allowed to solidify at 37°C. Embedded EBs were subsequently cultured in neural expansion medium containing 50% DMEM/F12, 50% Neurobasal medium, 0.5× N2 supplement, 0.5× B27 supplement without vitamin A, 2 mM GlutaMAX, 2.5 ng/ml human insulin, 0.5% non-essential amino acids (Life Technologies), and 25 nM beta-mercaptoethanol (Life Technologies). The tissue droplets were cultured in stationary condition in 6 cm suspension dishes for 4 days, followed by being transferred to an orbital shaker (Unimax-1010, Heidolph Brinkmann) rotating continuously at 75 rpm, in neural maturation medium containing 50% DMEM/F12, 50% neurobasal medium, 0.5× N2 supplement, 0.5× B27 supplement with vitamin A (Life Technologies), 2 mM GlutaMAX, 2.5 ng/ml human insulin, 0.5% non-essential amino acids, and 25 nM beta-mercaptoethanol.

### Viral infections

HSV-1 strain F stocks were propagated in Vero (African green monkey kidney) cells, with the Dulbecco’s modified Eagle’s medium (DMEM) supplemented with 2% Fetal Bovine Serum (FBS). The propagation was terminated when the 90% cells appeared the cytopathic effect. The virus stocks were harvested by repeatedly freezing and thawing the supernatants and cells for three times followed by filtering by the 0.22 μm filter, and stored in -80°C before using. The titers of HSV-1 stocks were determined by the plaque assay in Vero cells. Briefly, Vero cells were seeded in 6-well plates and incubated with a series of 10-fold virus dilutions for 2 hours. Then cells were overlaid with 2 ml/well of DMEM (Gibco) containing 1.2% methylcellulose, 10% (v/v) FBS, 1% penicillin-streptomycin solution (Invitrogen). Plates were incubated at 37°C and 5% CO_2_ for 5**–**6 days. Cells were fixed with 0.4% paraformaldehyde and stained with 0.5% crystal violet solution. HSV-1 strain F stocks were maintained at a stock concentration of 10^7^ pfu/mL.

Infection of monolayer neuronal differentiation model: the hiPSCs, hiPSC-derived NSCs, and NSCs-derived neurons were infected with cell-free HSV-1 with MOI of 0.2 or 2. The inocula were removed after two hours of infection. Then the cells were washed twice with preheated medium and then were cultured with fresh medium for 24 hours.

Infection of 3D brain organoid model: the cerebral organoids at different developmental stage were infected with the low or high titer of HSV-1(Low HSV-1:1000 pfu/organoid; High HSV-1:100000 pfu/organoid) in the culture medium for three days.

### Histology and immunofluorescence

Immunohistofluorescence analysis was performed as a previously described method[25]. The cells were fixed with 4% paraformaldehyde for 15 min and blocked with goat serum for 1 hour. Blocking and permeabilization was performed using 0.3% Triton X-100 and 3% normal donkey serum in PBS for 20 min. Primary antibodies were then incubated at 4°C overnight. Subsequently, the cells were incubated with the secondary antibodies at the following concentrations: Alexa Fluor 488 Goat anti-Mouse (1:200 dilution; Life Technologies), Alexa Fluor 568 Goat anti-Mouse (1:200 dilution; Life Technologies), Alexa Fluor 488 Donkey anti-Rabbit (1:200 dilution; Life Technologies) and Donkey anti- Rabbit (1:200 dilution; Life Technologies). The nuclei were counterstained with DAPI (Life Technologies). The images were taken using an Olympus microscope, Japan. Image J software (NIH, MD, USA) was used to merge images and to adjust the brightness or contrast uniformly. Detailed antibody information was described in Table 1.

**Table 1:** Key resource table.

Antibodies	Source	Identifier
Nanog	Santa Cruz	sc-5279
Oct-3/4	R&D	AF-1997
NeuN	Millipore	ABN78
MAP2	Abcam	ab11267
TUJ	Abcam	ab7751
SOX2	Boster	BA3292
NESTIN	Invitrogen	14984382
ISL1	Invitrogen	MA5-15515
GFAP	Invitrogen	MA5-12023
PAX6	Cell Signaling Technology	60433
Iba1	ABclonal Technology	A12391
CD11b	ABclonal Technology	A1581
HSV1 gE	Abcam	Ab6510

For section immunofluorescence, brain organoids were isolated from Matrigel, fixed in 4% (w/v) paraformaldehyde in PBS. The 8 μm thick sections from brain organoids were obtained with a cryostat (Leica). Images were captured on a Leica TCS SP8 STED confocal microscope equipped with a LAS X software. For quantification of protein distribution, the same laser intensities detector sensitivity and amplification values and offset were used for all micrograph acquisitions. The expression of nuclear and cytosolic protein were calculated by dividing the integrated optical density (IOD) with the total area of the nucleus or cytoplasm of the same cell. Moreover, each IOD/area value was calculated by subtracting background IOD/area value from the directly measured IOD/area value[26]. Images were prepared using ImageJ software (NIH, MD, USA).

### Real-time PCR

Total mRNAs were isolated from the human cerebral organoids or cells using Trizol reagent, then cDNA was synthesized using AccuPower RT-PCR PreMix (Bioneer). The qRT-PCR was performed using SYBR Green Real-time PCR Master Mixes (Invitrogen) under the following reaction conditions (35 cycles): denaturation at 95°C for 1 min, annealing at 58°C for 30 s, and extension at 72°C for 30 s. Primer sequences were described in Table 2. The expression levels were normalized relative to the expression of the housekeeping gene GAPDH[27] using the comparative Ct–method 2^−ΔΔCt^.

**Table 2:** Sequences of DNA primers for Real-time PCR in this study.

Gene	Forward Primer	Reverse Primer
SOX2	AAAATCCCATCACCCACAGCAA	AAAATAGTCCCCCAAAAAGAAGTCC
NESTIN	AGCGTTGGAACAGAGGTTGGAG	GGCTGAGGGACATCTTGAGGTG
ISL1	TCTTGCTGAAGCCGATGC	TGTTTGAAATGTGCGGAGTG
TUJ	TGATGCGGTCGGGATACTC	TGGGCCAAGGGTCACTACAC
MAP2	CAGGAGACAGAGATGAGAATTCC	CAGGAGTGATGGCAGTAGAC
Iba1	GAGATCAACAAGCAATTCCTA	ATCAATATCGCCATTTCCATTA
CD11b	CCAGAGAATCCAGTGTGA	GTTATGCGAGGTCTTGATG
GFAP	ACTGGCAGAGCTTGTTAGTG	AGTGACAGGAAGAGGTGAGA
IL-10	TGGAGCAGGTGAAGAATG	TCTATGTAGTTGATGAAGATGTC
IL-4	CCTCTGTTCTTCCTGCTA	AGATGTCTGTTACGGTCAA
TNF-α	GTGAGGAGGACGAACATC	GAGCCAGAAGAGGTTGAG
IL-6	TGAGAGTAGTGAGGAACAAG	CGCAGAATGAGATGAGTTG
GAPDH	GGACCTGACCTGCCGTCTAG	GTAGCCCAGGATGCCCTTGA

### Flow cytometry

For the apoptosis analysis, cells were stained with Annexin V-FITC/PI Apoptosis Detection Kit according to the manufacturer’s instructions. The apoptotic cells were detected by BD FACS Calibur™ (BD Biosciences, San Jose, CA, USA).

### RNA-Seq and data analysis

The hiPSC-derived NSCs were infected with HSV-1 at MOI of 0, 0.2, and 2. The inocula were removed after two hours of infection. Then the cells were washed twice with preheated medium, and then were cultured with fresh medium for 24 hours. Cells were harvested after 72 hours, and the RNAs was extracted. RNA sequencing was performed using an Illumina NextSeq 6000 with an average of 20 million reads per run. The GO (Gene Ontology) and KEGG (Kyoto Encyclopedia of Genes and Genomes) pathway enrichment analysis were performed using DAVID (https://david.ncifcrf.gov/). In order to get astringent DEG data set, only DEG with ≥ 1.5-fold change was used for GO analysis and KEGG pathway enrichment analysis. The Seqhealth Technology Company Limited supplied mRNA-seq technical support and data analysis. The transcriptomics data have been submitted to the Sequence Read Archive with the database identifiers PRJNA560001.

### Statistical analysis

Statistical analysis of data was expressed as means ± SEM. Student’s t tests were applied to data with two groups. ANOVA analyses were used for comparisons of data with greater than two groups. In all the analyses, group differences were considered statistically significant at p < 0.05 (*p < 0.05, **p < 0.01). Sample sizes were indicated in the Figure legends.

## Results

### HSV-1-infected NSCs exhibited increased cell apoptosis

**Fig 1:**
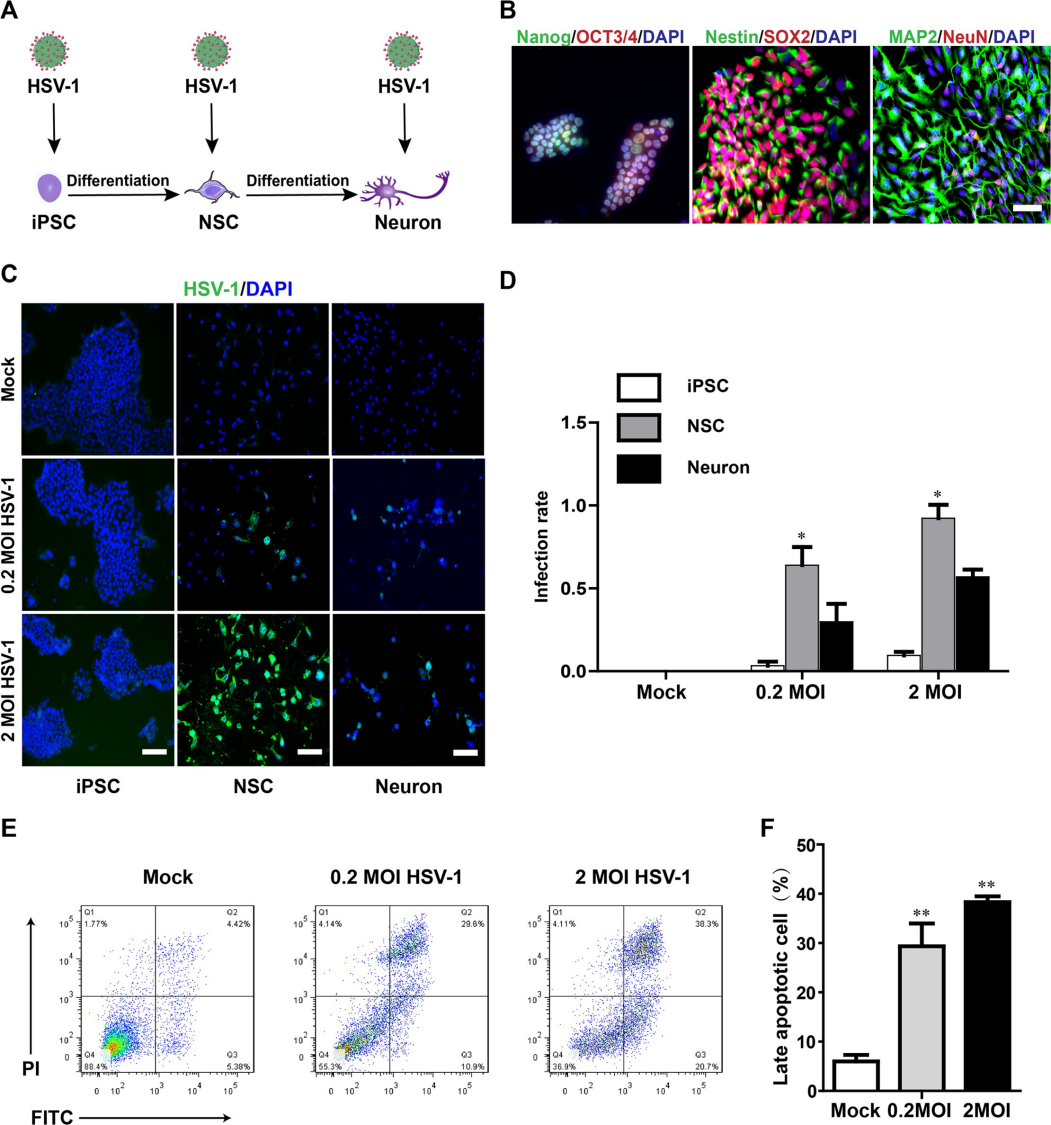
HSV-1-infected NSCs exhibited increased cell apoptosis. (A) Schematic representation of the experimental pipeline used in the differentiation process of hiPSC-derived neurons. (B) Confirmation of the characteristic expression of hiPSCs, hiPSC-derived NSCs, and NSC-derived neurons. Immunolabeling of SOX2 and Nestin in hiPSC-derived NSCs at the day 7; NSCs were further differentiated into neurons illustrated using MAP2 and NeuN immunofluorescence after the day 21 of neural induction. Scale bars represent 50 μm. (C) Sample images of hiPSCs, NSCs and neurons 24 hours after infection with HSV-1, immunostained for HSV1 gE envelop protein (green) and DAPI (blue). The images were taken using an Olympus microscope. Scale bars represent 100 μm. (D) Quantification of infection efficiency for different cell types, including hiPSCs, hiPSC-derived NSCs, and NSC-derived neurons. Data represent the mean ± SEM. *p < 0.05 by ANOVA (n = 3 per sample). (E) Flow cytometry analysis showed the apoptotic NSCs after 24 hours treatment with or without HSV-1 infection (0.2 MOI or 2 MOI) for 2 hours. (F) Bar graphs showed the percentage of late apoptotic cells. Data represent the mean ± SEM. *p < 0.05, **p < 0.01 by ANOVA (n = 3 per sample).

To identify the direct target cells of HSV-1 infection in the human neural lineage, we exposed hiPSCs, hiPSC-derived NSCs, and NSC-derived mature neurons to HSV-1 infection. The infection was performed at different multiplicity (0.2 MOI or 2 MOI), and the medium containing virus inoculum was removed after a 2-hour incubation period. Schematic representation of the experimental pipeline used in the differentiation process of hiPSC-derived neurons was shown in Fig 1A. After 7 days of neural induction from hiPSCs, the NSCs expressed high levels of SOX2 and Nestin, and the mature neurons which expressed NeuN and MAP2 were observed from NSCs after 21 days of differentiation (Fig 1B). The infection rates were then quantified 24 hours later using immunofluorescence staining against an anti-HSV-1 envelope antibody. HSV-1 glycoprotein E envelop protein immunostaining exhibited the characteristic weak speckled cytoplasmic and strong perinuclear staining pattern in infected Vero cells (S1A Fig). The hiPSCs could be infected by HSV-1, but the infection was only limited to a fraction of cells at the colony edge (S1B Fig). The mature neurons differentiated from NSCs also exhibited lower levels of infection under this condition (Fig 1C). The NSCs were highly susceptible to HSV-1 infection in a dose-dependent manner, with the transduction rate of 63.6% at 0.2 MOI of HSV-1 and 92.3% of the cells at 2 MOI HSV-1(Fig 1D). Together, these results indicated that hiPSC-derived NSCs, a constitutive population of the developing embryonic brain, were more permissive and acted as direct cells target of HSV-1 in the differentiation process of hiPSC-derived neurons.

We next determined the potential impact of HSV-1 infection on hiPSC-derived NSCs. Interestingly, the analysis of flow cytometry (Fig 1E and 1F) showed the apoptotic ratio remarkably increased following HSV-1 infections in a dose-dependent manner (26.34 ± 3.11 for 0.2 MOI group vs 8.23 ± 1.02 for control group, p = 0.0054; 42.66 ± 4.57 for 2 MOI group vs 8.23 ± 1.02 for control group, p = 0.0018).

The RNA-seq was also performed to guide the investigation of the regulatory effects of HSV-1 on the signaling pathways. The KEGG pathway enrichment analysis revealed that gene networks associated with apoptosis were the most significantly upregulated biological processes in HSV-1-infected NSCs (S2A Fig). Consistent with the result in Fig 1E and 1F, the heat map (S2B Fig) also showed that the HSV-1 infection promoted scaled mean expression levels of upregulated genes involved in apoptosis in a dose-dependent manner in NSCs. Therefore, HSV-1 infection of NSCs led to inhibiting the growth of this cell population, which was due, at least partially, to the induction of cell apoptosis.

### HSV-1-infected NSCs exhibited dysregulated neural differentiation

**Fig 2:**
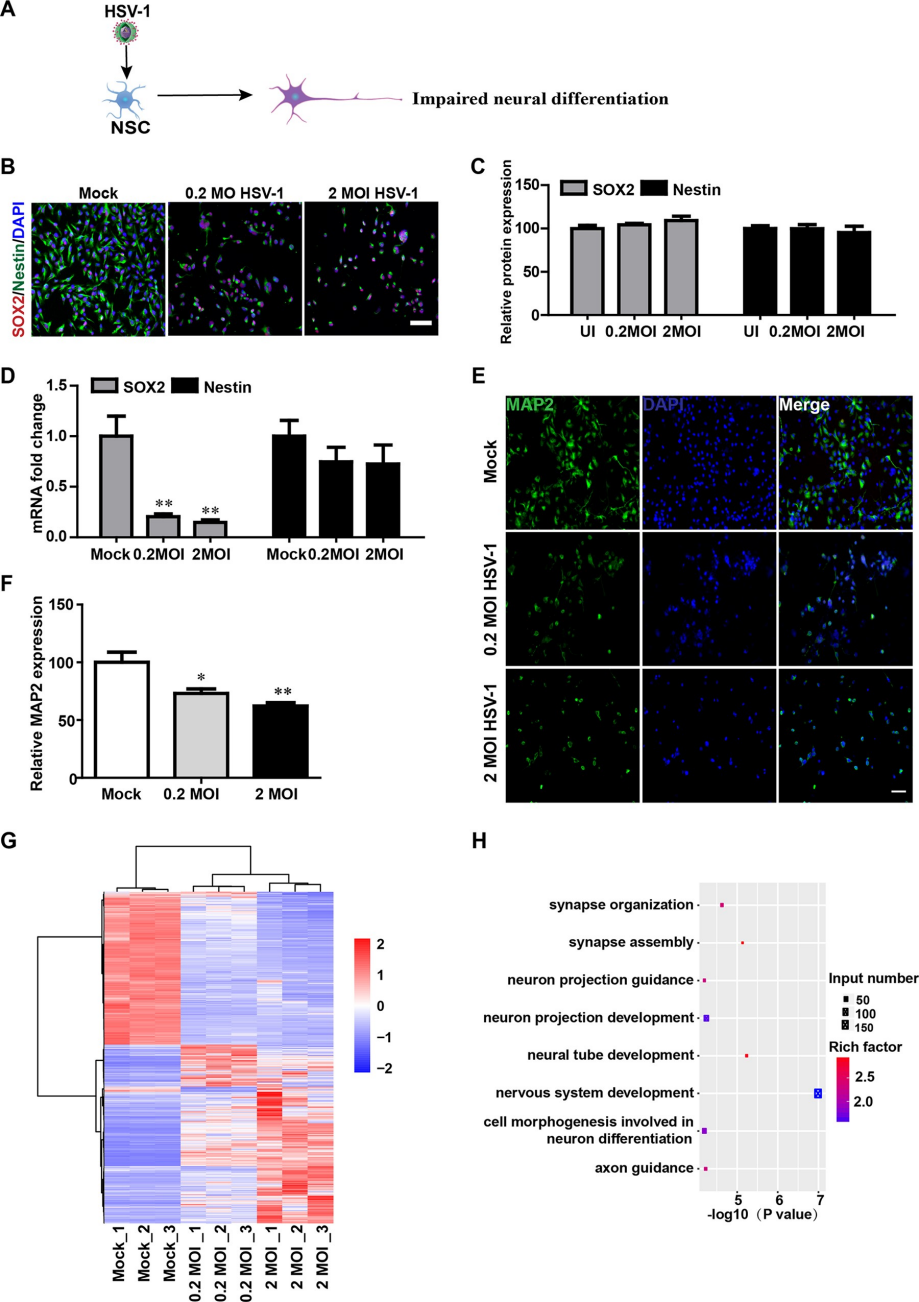
HSV-1-infected NSCs exhibited dysregulated neural differentiation. (A) Schematic diagram depicting the effect of HSV-1-infection on the NSCs in the term of neuronal differentiation. (B) Sample images of NSCs after 24 hour infection without or with HSV-1(0.2 MOI or 2 MOI) for 2 hours, immunostained for NSCs markers Nestin (green) and SOX2 (red) and DAPI (blue). The images were taken using an Olympus microscope. (C) Quantification of Nestin and SOX2. (D) Detection of the mRNA expression of SOX2 and Nestin (n = 3). (E) The expressions of MAP2 were identified by immunofluorescence staining, and the quantifications (F) for the percentage of MAP2+ were shown using Image J. Data represent the mean ± SEM. *p < 0.05, **p < 0.01 by ANOVA (n = 3 per sample). (G) Unsupervised hierarchical clustering of genes differentially expressed in NSCs on the basis of infection with or without HSV-1 (0.2MOI, 2MOI). (H) Gene Ontology (GO) biological process groups enriched in genes down-regulated in NSCs infected with the HSV-1(0.2 MOI), compared with the NSCs.

Previous report[28] demonstrated that HSV-1 infection impairs the growth and proliferation of mouse NSC proliferation and their neuronal differentiation. The schematic diagram (Fig 2A) depicted the effect of HSV-1-infection on the impaired neural differentiation. In our study, the HSV-1 infection did not alter the expression of NSC markers SOX2 and Nestin (Fig 2B and 2C), which are involved in cell proliferation and fate decision. However, the RNA expression of SOX2 was significantly reduced in the HSV-1 infected NSCs in a dose-dependent manner, whereas the Nestin RNA level was decreased with no statistical significance (Fig 2D).

We then determined whether HSV-1 infection could impair the ability of neural differentiation of NSCs. The hiPSC-derived NSCs were infected with or without HSV-1(0.2 MOI or 2 MOI) for 2 hours, and then further differentiated into neurons after 21 days of neural induction. At 21 days post-infection, we found that the immunoreactivity for the neuronal marker microtubule associated protein 2 (MAP2) was progressively decreased in the HSV-1- infected NSCs in a dose-dependent manner (Fig 2E and 2F), thus suggesting that HSV-1 infection markedly altered the neurogenic differentiation, at least, impaired the neuronal differentiation. In addition, our results in Fig 2A and 2D revealed that increasing MOI resulted in fewer total cells and caused profound morphological changes in NSCs. This type of cell change has also been shown in the previous article[29] which demonstrated that HSV-1 infection resulted in NSCs death and multicellular structures which stained strongly positive for amyloid fibrils, reminiscent of the hallmark features of Alzheimer’s disease within these structures. In our study, the infected NSCs formed large multicellular structures which retained neurite extensions. Overall, our results showed the cell death and multicellular structures, suggesting a neuropathological phenotype that may imply neurological diseases within these structures.

The RNA-seq was also performed to guide the investigation of the regulatory effects of HSV-1 infection on the signaling pathways. Gene expression profiling of HSV-1-infected NSCs showed robust differences (Fig 2G and S1 Table). The principal component analysis (PCA) revealed that uninfected NSCs, 0.2 MOI HSV-1-infected NSCs, and 2 MOI HSV-1-infected NSCs were separated (S2C Fig). GO enrichment analysis of the differentially expressed genes in uninfected and 0.2 MOI HSV-1-infected NSCs showed highly enriched categories related to synapse organization, synapse assembly, neural tube development, nervous system development, axon guidance, cell morphogenesis involved in neuron differentiation, neuron projection guidance and neuron projection development (Fig 2H). GO enrichment analysis of differentially expressed genes in uninfected and 0.2 MOI HSV-1-infected NSCs showed highly enriched categories related to neurogenesis and neuronal differentiation.

To further evaluate the gene associations among the enriched pathways related to the neurogenesis and neuronal differentiation related to synapse organization, synapse assembly, neural tube development, nervous system development, axon guidance, cell morphogenesis involved in neuron differentiation, neuron projection guidance and neuron projection development based on the GO analysis, these genes were mapped to form the key gene networks within the STRING database. We found that about 384 genes derived independently in each enriched pathway were highly correlated (S2D Fig). Thus, by comparing gene expression profiles between NSCs and 0.2 MOI HSV-1-infected NSCs, we identified that the gene enrichment in the processes related to the neurogenesis and neuronal differentiation were inhibited. The HSV-1 infection could be responsible for accelerating the risk of the dysregulated neural differentiation.

### The neuroepithelial buds and cerebral organoids recapitulated early stages of human fetal brain development

Native brain development exhibits an orderly sequence of processes that involve the formation of specific brain regionalization, hierarchical cortical structure, and neural differentiation. Cerebral organoids develop through intrinsic self-organizing processes upon timely application of components and cultural environments resembling the specific architecture and functions of the real brain[30].

**Fig 3:**
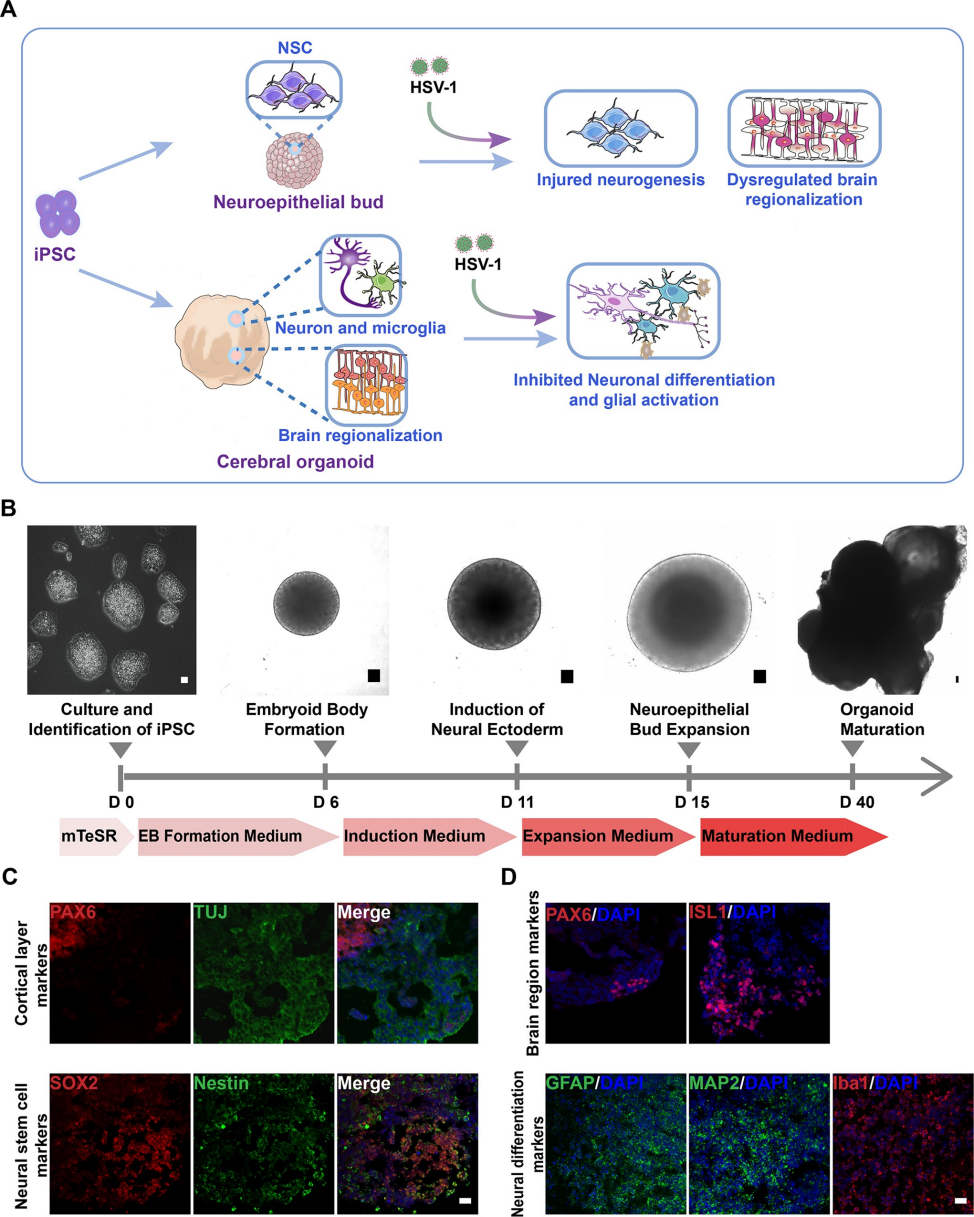
The neuroepithelial buds and cerebral organoids recapitulated early stages of human fetal brain development. (A) Schematic diagram depicting the effect of HSV-1-infection on the neuroepithelial buds and brain organoids in the areas of modelling the pathological features of developing human fetal brain, including injured neurogenesis, impaired neuronal differentiation, abnormal microglial activation, and dysregulated brain regionalization. (B) Schematic diagram of the neuroepithelial bud and cerebral organoid method and timing. Scale bars: 50μm. (C) The immunofluorescence staining for PAX6, TUJ, Nestin, and SOX2 in the neuroepithelial buds at the day 18. Scale bars: 25 μm. (D) Expressions of the specific brain regions markers (forebrain, PAX6; hindbrain ISL1) in brain organoids at the day 18; the cerebral organoids derived from hiPSCs at the day 45 showed the diverse neuron subtypes, including the mature neurons (MAP2), the astrocyte marker (GFAP), and microglia markers (Iba1). Scale bars: 25 μm. The images were taken Images were captured on a Leica TCS SP8 STED confocal microscope.

The schematic diagram (Fig 3A) depicted the effect of HSV-1-infection on the neuroepithelial buds and brain organoids. In brief, the neuroepithelial buds and the cerebral organoids mimicked various aspects of in vivo human brain development to produce interacting NSCs, neurons, and microglia. Our findings supported that HSV-1 infection led to the pathological process of neurodevelopmental disorder, such as the injured neurogenesis, impaired neuronal differentiation, abnormal microglial activation, and dysregulated brain regionalization. To generate neuroepithelial buds and human cerebral organoids, we followed the protocol described by Lancaster and Knoblich[23].The EBs formed by hiPSCs were suspended in Matrigel, and then the mixture transferred to an orbital shaker to enhance nutrient exchange. The Neuroepithelial buds were formed during the active period of proliferation of neurogenesis (from the day 15). At the end of the neural induction stage of our cerebral organoid-development protocol, cerebral organoids formed complex heterogeneous tissues (up to 4 mm in diameter) (Fig 3B). Next, we examined the detailed features of diverse cortical neuron subtypes, specific brain regionalization, and cortical organization in the cerebral organoids. Each neural-tube-like structure in the neuroepithelial buds develops independently to form one cortical structure, producing a variety of cell types including NSCs and neurons, which organize into distinct ventricular zone (VZ) and cortical plate (CP) layers. We found that cerebral organoids contained a well-defined VZ and CP layers. Specifically, the majority of the sites showed PAX6 positive (dorsal forebrain marker NSC marker) cells on the interior of the neural-tube-like structures, surrounded by TUJ positive (newborn neuron marker) cells on the outer edge of the structures. The NSC markers Nestin and SOX2 were revealed effective neural induction in neuroepithelial buds on day 18 (Fig 3C). The early brain regionalization during brain organoid development was identified by staining for specific forebrain marker (PAX6) and hindbrain markers (ISL1), indicating the various brain regions developed along the exterior in brain organoids (Fig 3D). Furthermore, the mature neurons (MAP2), the astrocyte marker (GFAP) and the microglia markers (Iba1) were all expressed in the cerebral organoids (Fig 3D). Taken together, these results demonstrated the efficient organization and formation of the neuroepithelial buds and cerebral organoids that recapitulated the key features in the developing human fetal brain at early stages.

### HSV-1 infection impaired neurogenesis in neuroepithelial buds

**Fig 4:**
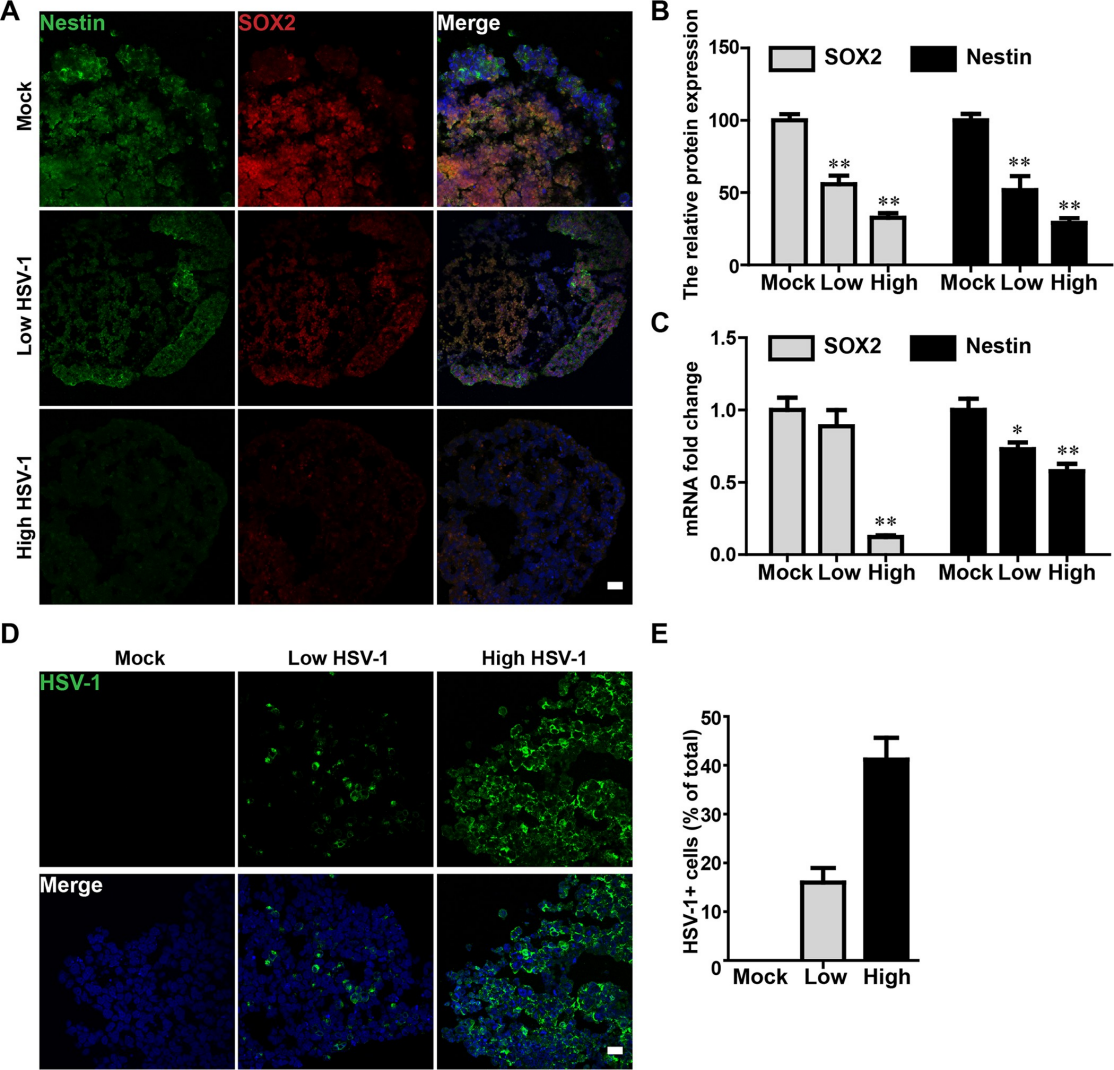
HSV-1 infection impaired neurogenesis in neuroepithelial buds. (A) The immunofluorescence staining for Nestin and SOX2 in the neuroepithelial buds at 18 days after 3 days with HSV-1 infection (Mock, Low infection, and High infection). Scale bars: 25 μm. The images were taken Images were captured on a Leica TCS SP8 STED confocal microscope. (B) Relative fluorescence intensity statistics of SOX2 and Nestin expressions were shown in different groups. Specifically, the expression of SOX2 was calculated by dividing the integrated optical density (IOD) with the total area of the nucleus, and the expression of Nestin was calculated by dividing the IOD with the total area of the cytoplasm. Images were prepared using ImageJ software (NIH, MD, USA). Data represent the mean ± SEM. *p < 0.05, **p < 0.01 by ANOVA (n = 3 per sample). (C) The mRNA expressions of Nestin and SOX2 were identified by RT-PCR. Data represent the mean ± SEM. *p < 0.05, **p < 0.01 by ANOVA (n = 4 per sample). (D) The immunofluorescence staining on the neuroepithelial buds was performed for detecting positive HSV-1 gE envelop protein at 18 day after 3 days with HSV-1 infection. Scale bars: 25 μm. (E) Bar graphs showing the percentages of HSV-1-positive cells in the neuroepithelial buds. Data represent the mean ± SEM from four experiments.

As seen above the effects of HSV-1 infections on fetal brain development are largely dependent on the HSV-1 viral load and target cells, we infected brain organoids with HSV-1 virus at different doses (Low HSV-1: 1000 pfu/organoid; High HSV-1: 100000 pfu/organoid) to explore the effects of HSV-1 infections on neurodevelopmental disorder. The organoids were infected with HSV-1 during the active period of proliferation of neuroepithelium and neurogenesis (from day 15). Next, the neurogenesis within HSV-1-infected neuroepithelial buds from EBs was investigated by immunofluorescence staining against SOX2 and Nestin on day 18. The data showed a significant decrease in the SOX2 and Nestin expressions, suggesting the injured neurogenesis following HSV-1 infection in a dose-dependent manner (Fig 4A and 4B). Similarly, the HSV-1 infected neuroepithelial buds displayed a low level of SOX2 and Nestin mRNA expression in both dosage groups by real-time PCR assay (Fig 4C). To be noted, the neuroepithelial buds showed significant inhibition of neurogenesis by the HSV-1 infection while the hiPSC-derived NSCs with HSV-1 infection did not reveal significant changes. In the HSV-1 infected neuroepithelial buds, we also tested the HSV-1 expression. The HSV-1 infection led to increased HSV-1 expression by immunofluorescence analysis in a dose-dependent manner (Fig 4D and 4E).

### Dysregulated the cortical layer and hindbrain populations in HSV-1-infected neuroepithelial buds

**Fig 5:**
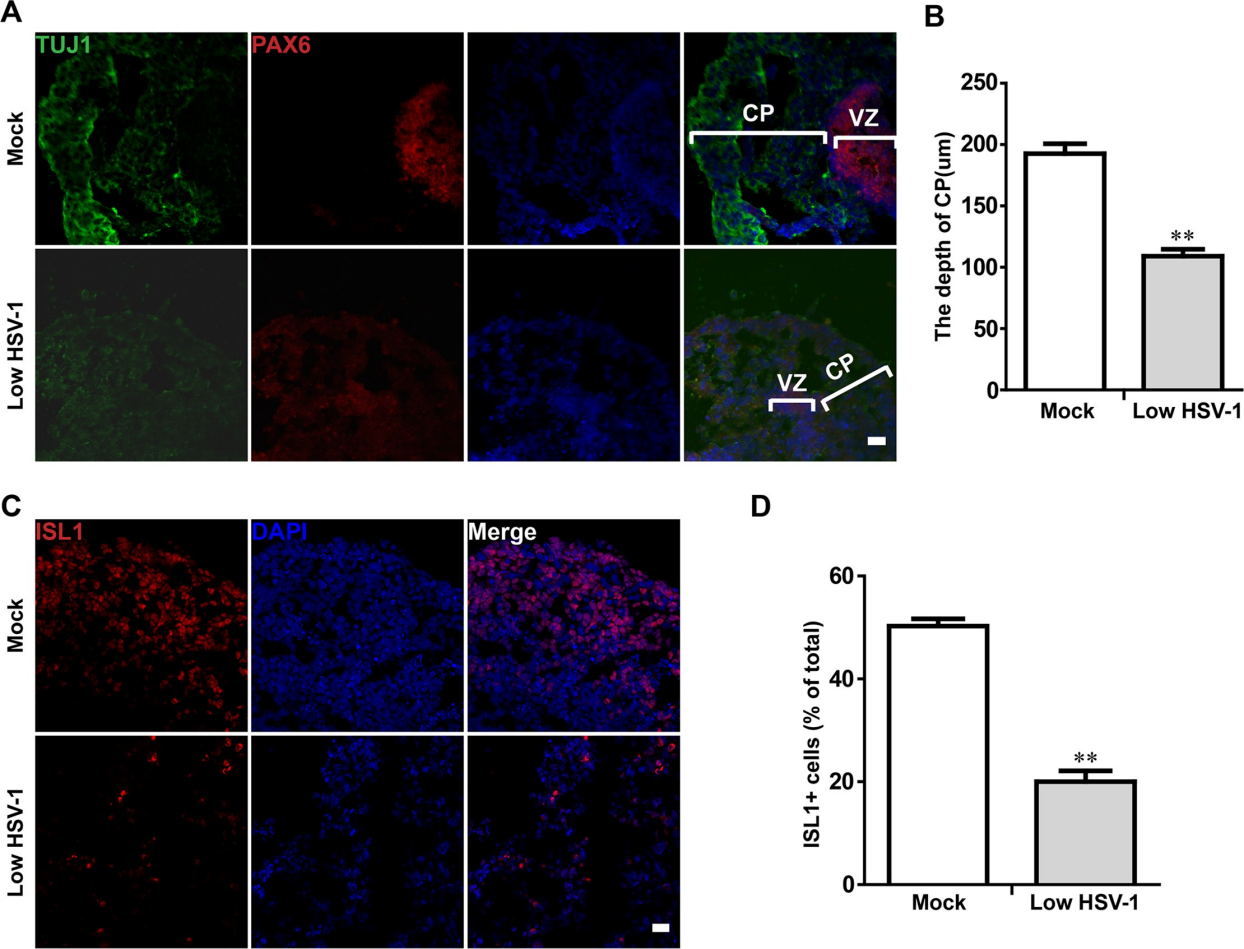
Dysregulated the cortical layer and hindbrain populations in HSV-1-infected neuroepithelial buds. (A) The dorsal NSC marker (PAX6) and newborn neuron marker (TUJ) were observed in the brain cerebral organoids at the 18 day after 3 days with HSV-1 infection (Mock, Low infection). Scale bars: 25 μm. The images were taken Images were captured on a Leica TCS SP8 STED confocal microscope. (B) Comparison of depths of CP layer between mock- and HSV-1-infected neuroepithelial buds. (C) Immunofluorescence staining for the hindbrain marker (ISL1) in neuroepithelial buds at the day 18 after 3 days with HSV-1 infection. Scale bars: 25 μm. (D) Quantification of ISL1-positive cells in the presence or absence of HSV-1 infection. Data represent the mean ± SEM from four experiments. **p < 0.01 by Student’s t test.

We analyzed the organization of dorsal cortical regions within cerebral organoids. At Day 18, we found that neuroepithelial buds contained a distinct ventricular zone (VZ) and cortical plate (CP) layers (Fig 5A).To understand the change during these structures development of the HSV-1 infection, we measured the CP layer depth. Compared with control group, the CP layer in the HSV-1-infected neuroepithelial buds was statistically thinner (Fig 5B). These neuroepithelial buds after HSV-1 infection led to a considerable loss of TUJ-positive cells, suggesting a statistically significant decrease in neuron production.

As the development proceeded in neuroepithelial buds, we further examined the effects of HSV-1 infection on the specific brain regionalization. The HSV-1 infected cerebral organoids at the day 18 after 3 days with HSV-1 infection showed the decreased hindbrain marker ISL1 as compared to the untreated control by immunofluorescence analysis (Fig 5C and 5D).

### HSV-1 infection inhibited neuronal differentiation in HSV-1-infected cerebral organoids

**Fig 6:**
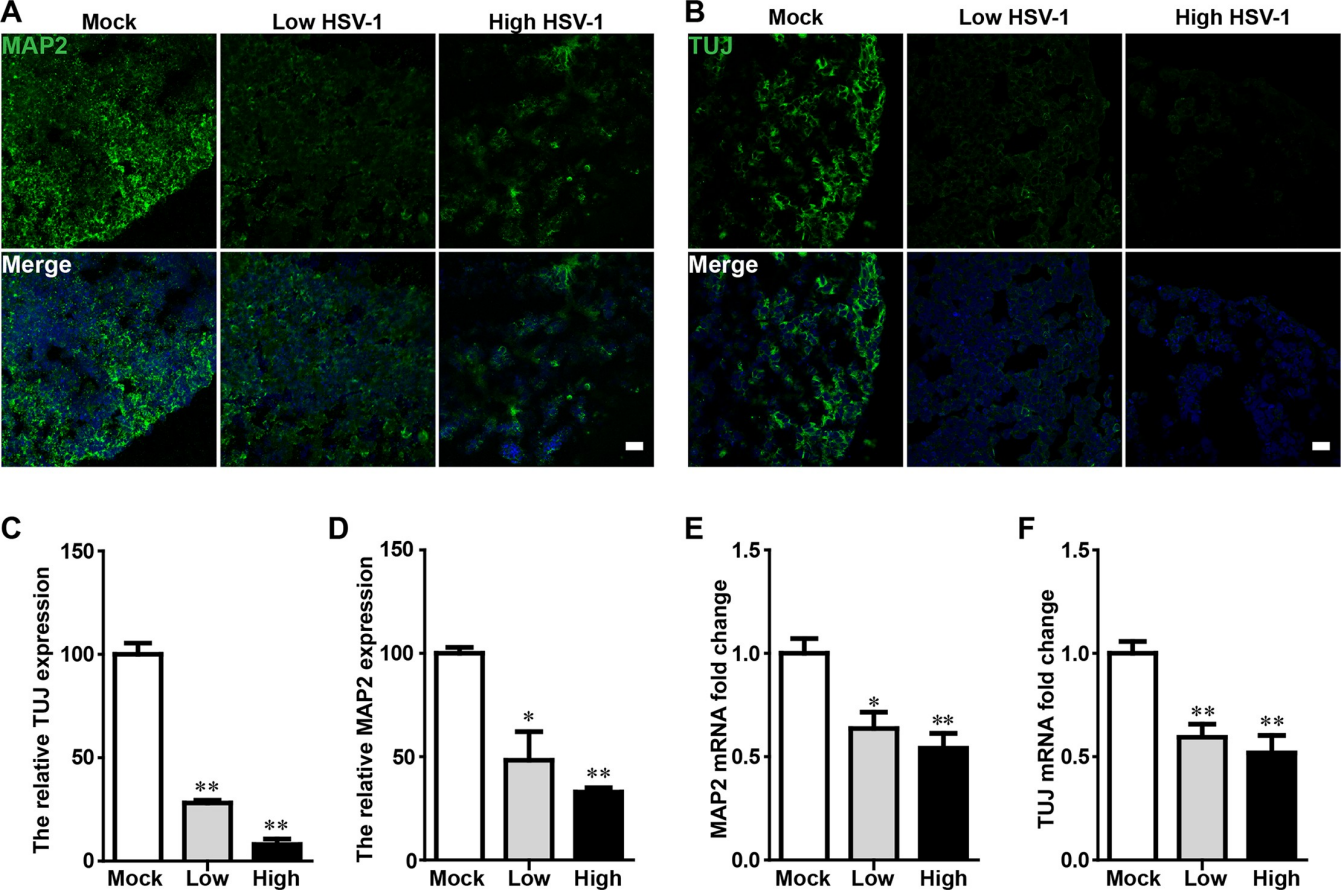
HSV-1 infection inhibited neuronal differentiation in HSV-1-infected cerebral organoids. (A and B) Immunofluorescence staining for the mature neuronal marker (MAP2) and early neurons marker (TUJ) in cerebral organoids at the day 45 after 3 days with HSV-1 infection. The images were taken Images were captured on a Leica TCS SP8 STED confocal microscope. Scale bars: 25 μm. (C and D) The relative fluorescence intensity statistics of MAP2 and TUJ expressions were shown in different groups. Data represent the mean ± SEM. *p < 0.05, **p < 0.01 by ANOVA (n = 3 per sample). (E and F) Validation by RT-PCR of MAP2 and TUJ in cerebral organoids at the day 45 for 3 days with HSV-1 infection (Mock, Low infection, and High infection). Data represent the mean ± SEM. *p < 0.05, **p < 0.01 by ANOVA (n = 4 per sample).

The process of neocortical development during human gestation involves neuronal subtype specification. The hiPSC-derived cerebral organoids offered a model to examine the specification of neural subtypes in human brain development. The specialized neural subtypes were examined to further explore the effects of HSV-1 infection on brain organoid development. On the day 45 after 3 days with or without HSV-1 infection, we examined the expression of neuron markers (MAP2 and TUJ). Cerebral organoids displayed markedly decreased MAP2 and TUJ expression after HSV-1 infection by immunofluorescence analysis (Fig 6A and 6B) and quantifications (Fig 6C and 6D) in a dose-dependent manner. The relative fluorescence intensity of MAP2 and proteins were decreased to 48.2% and 28.08%, respectively, in Low-dose group. In High-dose group, these values were further decreased to 32.9% and 8.02%. In addition, the mRNA expressions of MAP2 and TUJ in HSV-1-infected cerebral organoids were also examined by real-time PCR (Fig 6E and 6F), which were consistent with the results observed in immunofluorescence analysis. Specifically, when compared to untreated control, MAP2 mRNA expression was down-regulated by 0.63 folds and 0.54 folds in Low-dose group and High-dose group, respectively. Similarly, the TUJ mRNA expression was inhibited by 0.54 folds in Low-dose group and 0.51 folds in High-dose group. These observations demonstrated that HSV-1 infection inhibited neuronal differentiation. This results might help to understand various postnatal cognitive dysfunctions due to the neonatal HSV infection.

### HSV-1 infection promoted abnormal microglial proliferation and activation in cerebral organoids

**Fig 7:**
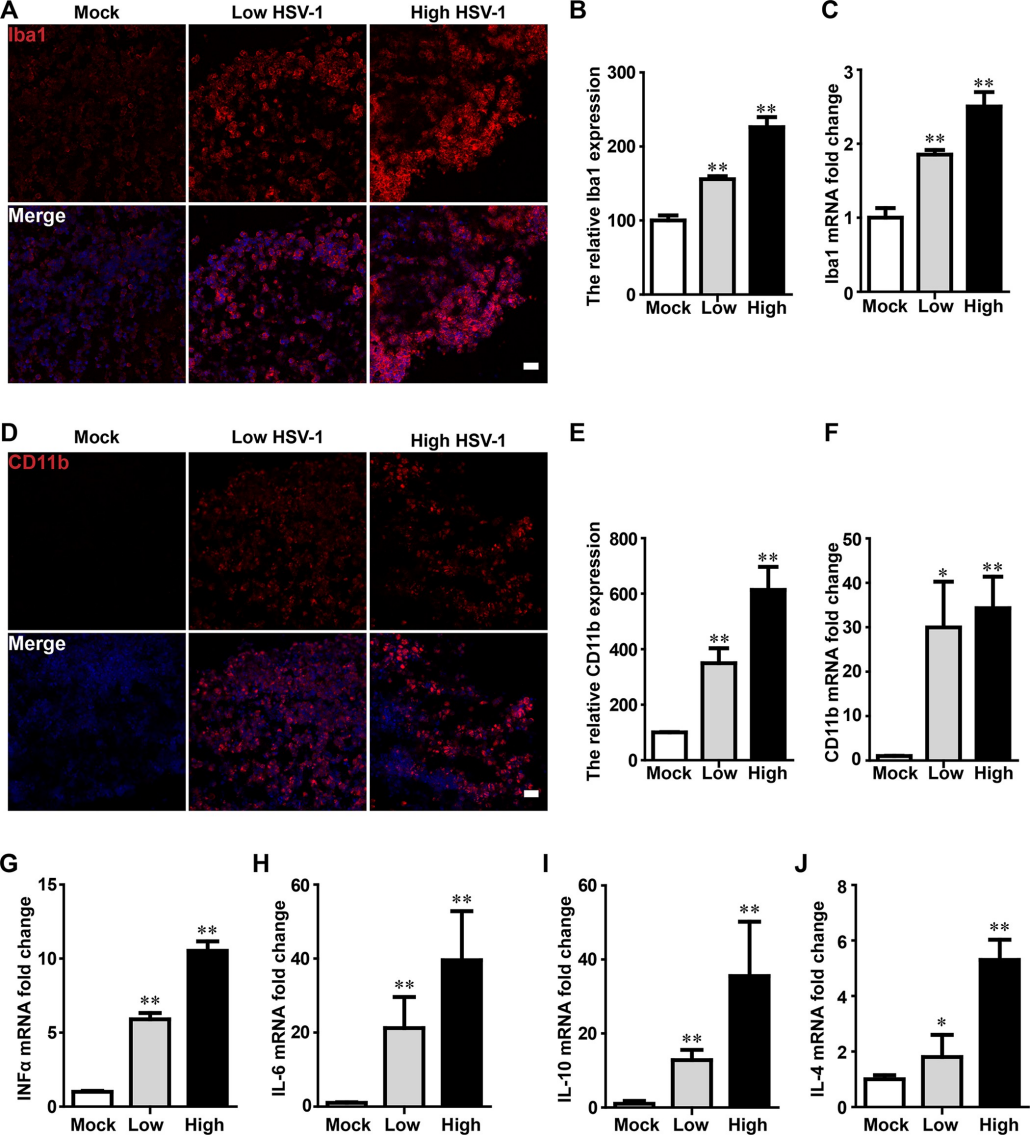
HSV-1 infection promoted abnormal microglial proliferation and activation in cerebral organoid. (A and D) Immunofluorescence staining for microglia markers (Iba1) and activated microglia (CD11b) in cerebral organoids at the day 45 after 3 days with HSV-1 infection. The images were taken Images were captured on a Leica TCS SP8 STED confocal microscope. Scale bars: 25 μm. (B and E) The relative fluorescence intensity statistics of Iba1 and CD11b expressions were shown in different groups. (C and F) Validation by RT-PCR of CD11b and Iba1 in cerebral organoids at the day 45 for 3 days with HSV-1 infection (Mock, Low infection, and High infection). Data represent the mean ± SEM. *p < 0.05, **p < 0.01 by ANOVA (n = 4 per sample). (G-J) The mRNA expressions were examined for the inflammatory cytokines (TNF-α, IL-6, IL-10, and IL-4) using RT-PCR in brain cerebral organoids at the day 45 after 3 days with or without HSV-1 infection. Data represent the mean ± SEM. *p < 0.05, **p < 0.01 by ANOVA (n = 3 per sample).

HSV-1 infection stimulated microglial proliferation by increasing Iba-1 -positive cell. The significant increase of Iba1 -positive cells were observed in organoids with HSV-1 infection by immunofluorescence analysis (Fig 7A) and quantifications (Fig 7B). The HSV-1 infection tended to increase fluorescence intensity of Iba1 protein in both Low-dose group (increased by 55.93%) and High-dose group (increased by 126.15%) as compared to the mock uninfected group. Consistently, HSV-1-infected cerebral organoids exhibited increased Iba1 mRNA expression (by 1.85 folds in Low-dose group and 2.5 folds in High-dose group) (Fig 7C). The activated microglia were detected with the CD11b -positive cells. The relative fluorescence intensity of CD11b protein were significantly different among three groups with dose response to HSV-1 infection. The CD11b expression was increased by 249.7% in low dose group and 513.6% in high dose group (Fig 7D and 7E) as compared to the mock group. In agreement with the protein expression, the mRNA expressions of CD11b (29.99 folds) in Low-dose group were higher in comparison to untreated control. In High-dose group, these values were further increased to 34.30 folds (Fig 7F). The results demonstrated that HSV-1 infection significantly promoted microglial proliferation and microglial activation.

Since the microglia have been reported to seriously influence the pathogenesis of the brain homeostasis due to the regulation of functional inflammatory, which strongly contribute to neurodevelopmental disorder. We found that an acute cytokine response can be assessed 36 hours after HSV-1 infection. As shown in Fig 7G–7J, brain organoids infected with HSV-1 exhibited increased mRNA expressions of the pro-inflammatory mediators (TNF-α and IL-6) and anti-inflammatory mediators inflammatory cytokines (IL-10, and IL-4). When compared to untreated control, the mRNA expressions of TNF-α, IL-6, IL-10, and IL-4 in Low-dose group, were increased by 5.90 folds, 21.54 folds, 12.83 folds, and 1.80 folds, respectively. In High-dose group, these the mRNA expression were further increased by 10.52 folds, 34.25 folds, 35.54 folds, and 5.30 folds, respectively.

### HSV-1 infection accelerated the astrocytes activation in neuroepithelial buds and cerebral organoid

**Fig 8:**
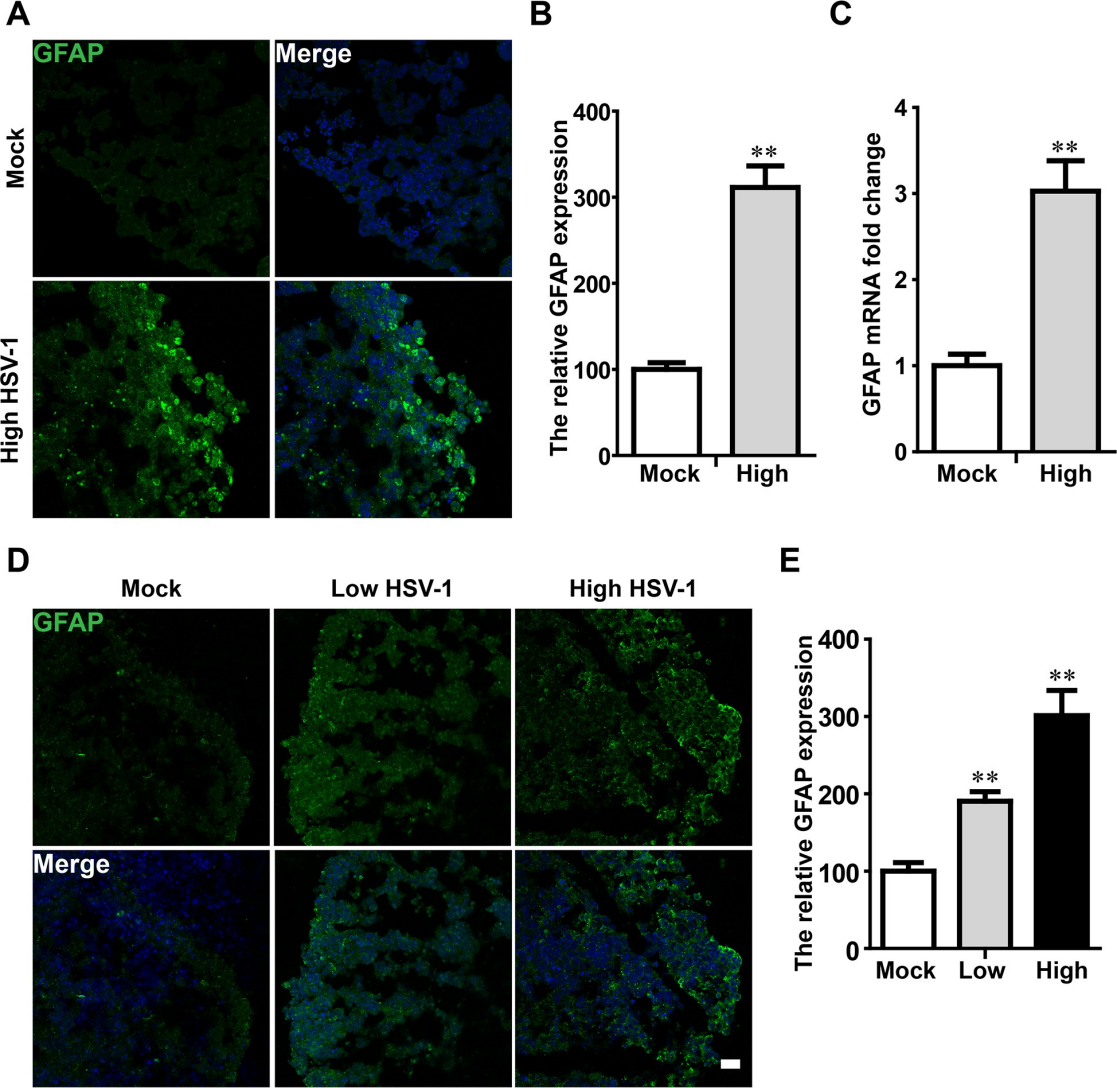
HSV-1 infection accelerated the astrocytes activation in neuroepithelial buds and cerebral organoids. (A) Immunofluorescence staining for the astrocyte marker (GFAP) in neuroepithelial buds at the day 18 after 3 days with HSV-1 infection. The images were taken Images were captured on a Leica TCS SP8 STED confocal microscope. (B) Relative fluorescence intensity statistics of GFAP expression were shown in different groups. (C) Detection of GFAP mRNA expression in neuroepithelial buds (n = 4). Data represent the mean ± SEM. **p < 0.01 by Student’s t test. (D) The sample images of immunostaining for GFAP in the cerebral organoids at the day 45 for 3 days with HSV-1 infection (Mock, Low infection, and High infection). Scale bars: 25 μm. (E) The relative fluorescence intensity statistics of GFAP expressions were shown in different groups. Data represent the mean ± SEM from three experiments. **p < 0.01 by ANOVA.

The extensive activation of astrocytes significantly generated in neuroepithelial buds and cerebral organoid with HSV-1 infection (Fig 8). On day 18 after 3 days with or without HSV-1 infection, the quantification of staining against astrocyte marker (glial fibrillary acidic protein, GFAP) (Fig 8A and 8B) was increased by 301% in High-dose group, suggesting the promoted the of activation astrocytes following HSV-1 infection in the neuroepithelial buds. Consistently, HSV-1-infected neuroepithelial buds exhibited increased GFAP mRNA expression (by 3.02 folds in High-dose group) (Fig 8C).

On day 45 after 3 days with or without HSV-1 infection, we examined the GFAP expression in the cerebral organoids. Similarly, the data in Fig 8D and 8E showed a significant increase in mean fluorescent intensity of GFAP expression in dose depend manner with 90% increase in low-dose group and 200% increase in high-dose group by immunofluorescence analysis.

## Discussion

HSV-1and HSV-2 are the two members of the herpesviridae family[9]. HSV poses a serious threat to pregnant women and their fetus. Beginning in the 1920s, a growing number of investigators have identified the critical roles of HSV infection in the abnormalities of the CNS.[13,14] Interestingly, HSV was detected in the cerebrospinal fluid of a premature infant, suggesting that HSV could get across the placental barrier and transmit across the blood-brain barrier in the developing human fetal brain[31]. There is accumulating evidence indicating that both of HSV-1 and HSV-2 tend to be neuro-invasive and become the underlying cause of life-long latent infection in the CNS[11]. In particular, the HSV-1 activation can lead to severe NDDs which cause later neuropsychiatric problems that persist into adulthood[32–34], including neuroinflammation, psychical disease, learning disabilities, intellectual disability, and the encephalitis[35] with the significant morbidity and mortality. Because so little is known about the permissive cell targets and potential impact of HSV-1 on human fetal brain development, with the limited access to fetal human brain tissue, the pathogenesis of HSV-1infection in human fetal brain development has not been completely understood. In this study, we have established in vitro neurodevelopmental disorder models to recapitulate HSV-1 infection-induced neurodevelopmental disorders.

Several mechanisms[36,37] may explain that prenatal processes in neurodevelopmental disorders might underlie early-age brain growth defects, such as abnormal cell proliferation and differentiation. For example, Donatella Li Puma et al.[28] reported that HSV-1 affects adult hippocampal neurogenesis by reducing NSC proliferation and their neuronal differentiation in mouse neurodevelopmental disorder model. One key finding from our analysis is that the HSV infection led to dysregulated neurogenesis in the fetal neurodevelopment. For neuroepithelial buds and brain organoids, the cell-cell and cell-ECM interactions in 3D environment allow cells to grow and expand in multiple directions and form specific niches that favor fate specification[38]. The cell signals generated in neuroepithelial buds and brain organoids are completely different from that in monolayer cell culture. We obtained conflicting results that HSV-1 infection impaired neurogenesis in NSCs. While the brain neuroepithelial buds showed a decreased level of Nestin (NSC marker) expression with HSV-1 infection, no significant changes were observed in Nestin expression in monolayer NSCs. This discordance between organoids and 2D monolayer cell culture has been widely found in the fields of development biology, disease modeling, and drug screening. For example, Hans Clevers and his colleagues revealed that the hemi-hepatectomy response (Afp, cell cycle gene) markers were highly expressed in the hepatocyte organoids compared to that in the monolayer hepatocyte culture[39].

Moreover, Leonardo D’Aiuto et al.[27] established 2D neuronal cells and 3D organoids derived from hiPSCs could recapitulate the immediate early gene ICP4 in the nuclei of MAP2-positive neurons, thus in further support our work that the permissiveness of central nervous system-like cultured cells to HSV-1. Thus, our study provided an encouraging proof of the conclusion that HSV-1 infection may contribute to neurodevelopmental disorders by targeting NSCs.

Previous studies[6] demonstrated that the major alterations included the alterations in cortical layer, hindbrain populations and impairments in neuron function in the offspring undergoing neurodevelopmental disorders. We also performed human cerebral organoids to study the effect of HSV-1 infection on the differentiation of specific brain regions in the process of brain development. And our study highlighted that HSV-1 infection on human cerebral organoids led to reduce the CP layer depth and the expression of hindbrain markers (ISL1). However, in the rodents models, some studies[40] found no change in total brain volume, but the relative volume of several regions was unilaterally increased or decreased observed in the offspring undergoing the neurodevelopmental disorders. Notably, a recent study demonstrated that HSV-1 infection induces the neuronal damage in memory formation-associated hippocampus tissue in rat organotypic hippocampal slice cultures[41], providing new insight into the roles of HSV-1 infection in affecting the multiple brain regions, including the cerebral cortex and hippocampus. Thus, our findings in HSV-1 infected human cerebral organoids raised the possibility that the HSV-1-induced dysregulated brain regionalization may occur in different stages of fetal brain development.

Other risk factors associated with neurodevelopmental disorder were also identified in this study. We found that the HSV-1 infection impaired the differentiation of specialized neural subtypes in brain organoids. The HSV-1-infected cerebral organoids at the day 45 inhibited neuronal differentiation, and stimulated microglial proliferation activation. Recent evidence[42] from clinical and preclinical investigations indicate that microglial dysfunction may be a critical cellular mechanism linked to brain development impairment, and microglial priming has been considered to underlie neuronal dysfunctions and aberrant postnatal behaviors in offspring. In addition, R. Jeroen Pasterkamp and his colleagues showed that the mesodermal-derived microglia could innately develop within a cerebral organoid model and display their characteristic ramified morphology[43]. This evidence provided new insights in understanding the neurodevelopmental disorder with microglial dysfunction and neural network function using the brain organoids. Remarkably, abnormal microglial activation indicating inflammation has been reported in the brain of patients with neurodevelopmental disorder. It is becoming more widely accepted that a dual role of microglia that depends on their phenotypes, classical activation (M1 phenotype) and an alternative activation (M2 phenotype)[44]. The M1 phenotype is primarily characterized by increased pro-inflammatory mediators such as INFα, TNF-α and IL-6. The M2 phenotype is primarily characterized by anti-inflammatory mediators such as IL-10, IL-4 and TGF-β.[36] However, we identified that the brain organoids infected with HSV-1 exhibited increased mRNA expressions of pro-inflammatory mediators (TNFα and IL-6) and anti-inflammatory mediators (IL-10 and IL-4). The rebalance of M1-M2 microglial phenotypes and pro-/anti-in-flammatory cytokines was associated with the reversal of neurodevelopmental disorder. M1-to-M2 phenotypic switch exhibited similar expression patterns in our HSV-1-infected organoids, providing new insights in understanding etiology of neurodevelopmental disorder.

In addition, with the same purpose of improving the human physiological relevance of in vitro brain models, brain organoid and brain-on-a-chip[45] represent two typical approaches based on the strategies of developmental biology and bioengineering, respectively. In contrast, brain-on-a-chip provides better capacity to reconstitute in-vivo neural microenvironments including heterocellular interactions, extracellular matrix and hemodynamics, in a deterministic manner. By elegantly combining the benefits of both developmental biology and bioengineering through a synergistic strategy, the brain organoid-on-a-chip[46] potentially offers a more faithful human-physiologically-relevant organotypic model to study prenatal neurophysiology and neuropathology in the future. In summary, our study provides in vitro neurodevelopmental disorder models of HSV-1 infection for studying the effect of HSV-1 on human brain development. The integrated findings of this study suggest that HSV-1 infection disrupted fetal brain development, including the injured neurogenesis, dysregulated forebrain and hindbrain populations, impaired neuronal differentiation, and abnormal microglial activation. Our strategy opens a new avenue for imitating the neuropathological features of fetal brain development with HSV infection, establishing the causal relationships that link HSV biology with the neurodevelopmental disorder-associated neuropathological changes.

## Supporting information

S1 Fig Related to Fig 1. (A-B) Sample images of Vero cells and hiPSCs after 24-hour infection without or with HSV-1 (2 MOI) for 2 hours, immunostained for HSV-1 gE envelop protein (green) and DAPI (blue). Scale bars represent 50 μm.
(TIF)

S2 Fig Related to Fig 1 and Fig 2. (A) Gene Ontology (GO) biological process groups enriched in genes down-regulated in NSCs infected with the HSV-1 (0.2 MOI), compared with the NSCs. (B) Heat map of apoptosis-related genes of RNA-seq dataset in HSV-1 infected NSCs. The apoptosis-related genes were obtained from http://www.genome.jp/kegg-bin/show_pathway?hsa04210/hsa:3708%09red/hsa:8503%09red/hsa:8793%09red/hsa:3725%09red/hsa:8797%09red/hsa:8794%09red/hsa:841%09red/hsa:843%09red/hsa:468%09red/hsa:330%09red/hsa:5414%09red/hsa:1521%09red/hsa:7124%09red/hsa:5604%09red/hsa:63970%09red/hsa:4616%09red/hsa:8743%09red/hsa:2353%09red/hsa:5170%09red/hsa:8739%09red/hsa:4914%09red/hsa:2081%09red/hsa:9451%09red/hsa:4792%09red/hsa:10018%09red/hsa:4790%09red/hsa:5366%09red/hsa:51807%09red/hsa:10912%09red/hsa:1647%09red/hsa:4000%09red/hsa:1514%09red/hsa:7277%09red/hsa:7157%09red/hsa:1147%09red/hsa:421.(C) PCA was performed based on transcriptomics data in the NSCs infected with or without HSV-1(0.2 MOI or 2 MOI) with a non-zero variance. (D) The key gene networks of the enriched pathways related to the neurogenesis and neuronal differentiation related to synapse organization, synapse assembly, neural tube development, nervous system development, axon guidance, cell morphogenesis involved in neuron differentiation, neuron projection guidance and neuron projection development based on the GO analysis. Search Tool for the Retrieval of Interacting Genes (STRING) was used to form key gene networks, the interactions with combined score ≥ 0.4 (medium confidence) were considered significant.
(TIF)

S1 Table List of Differentially Expressed Genes in uninfected NSCs, 0.2 MOI HSV-1-infected NSCs, and 2 MOI HSV-1-infected NSCs. (XLS)
