# Supplementary figures and images for: Herpes simplex virus type 1 infection leads to neurodevelopmental disorder-associated neuropathological changes
Source: PLoS Pathog. 2020 Oct 22;16(10):e1008899. doi: 10.1371/journal.ppat.1008899 (PMC7580908; doi:10.1371/journal.ppat.1008899)

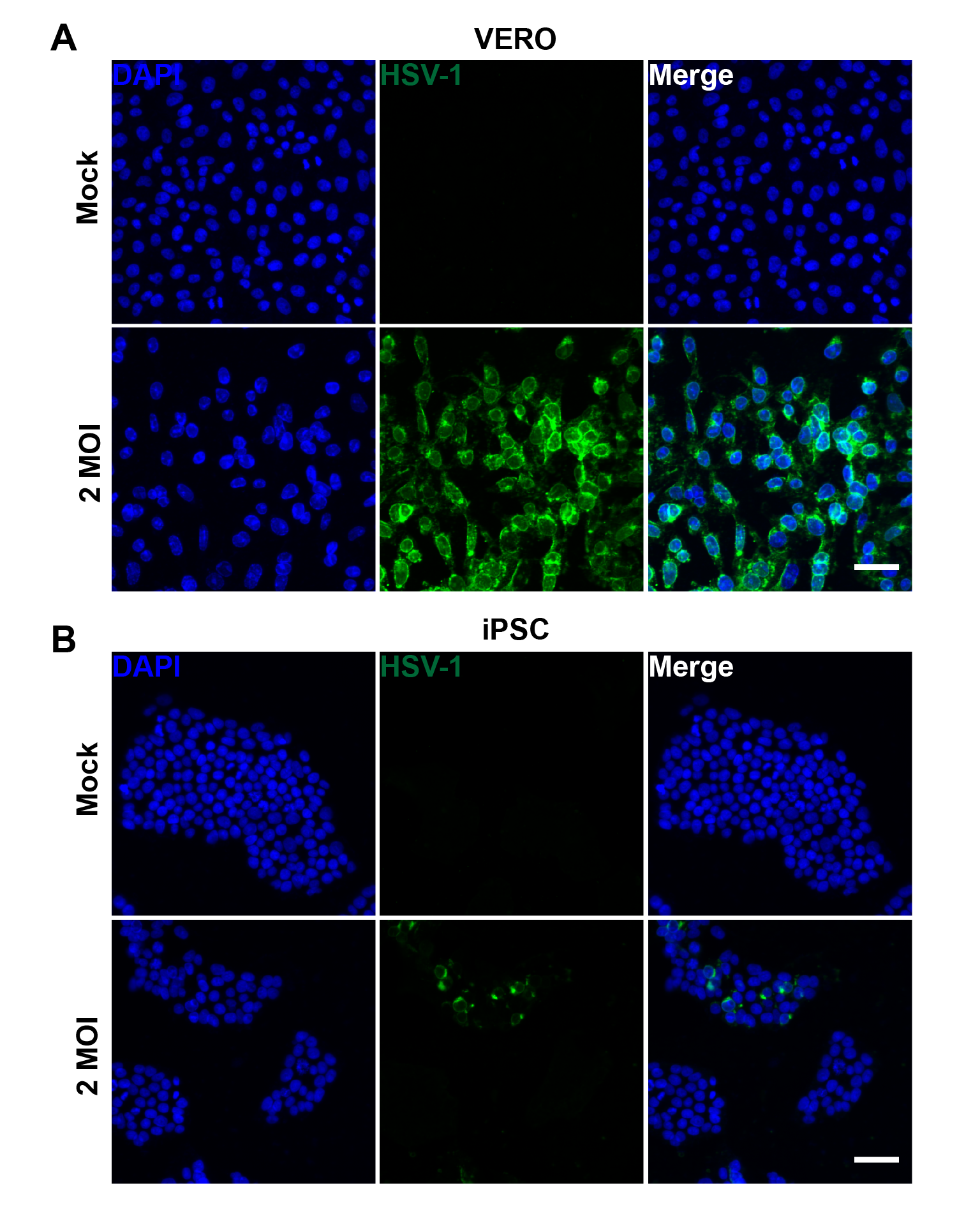

Supplement: S1 Fig — (A-B) Sample images of Vero cells and hiPSCs after 24-hour infection without or with HSV-1 (2 MOI) for 2 hours, immunostained for HSV-1 gE envelop protein (green) and DAPI (blue). Scale bars represent 50 μm. (TIF) [file ppat.1008899.s001.tif]

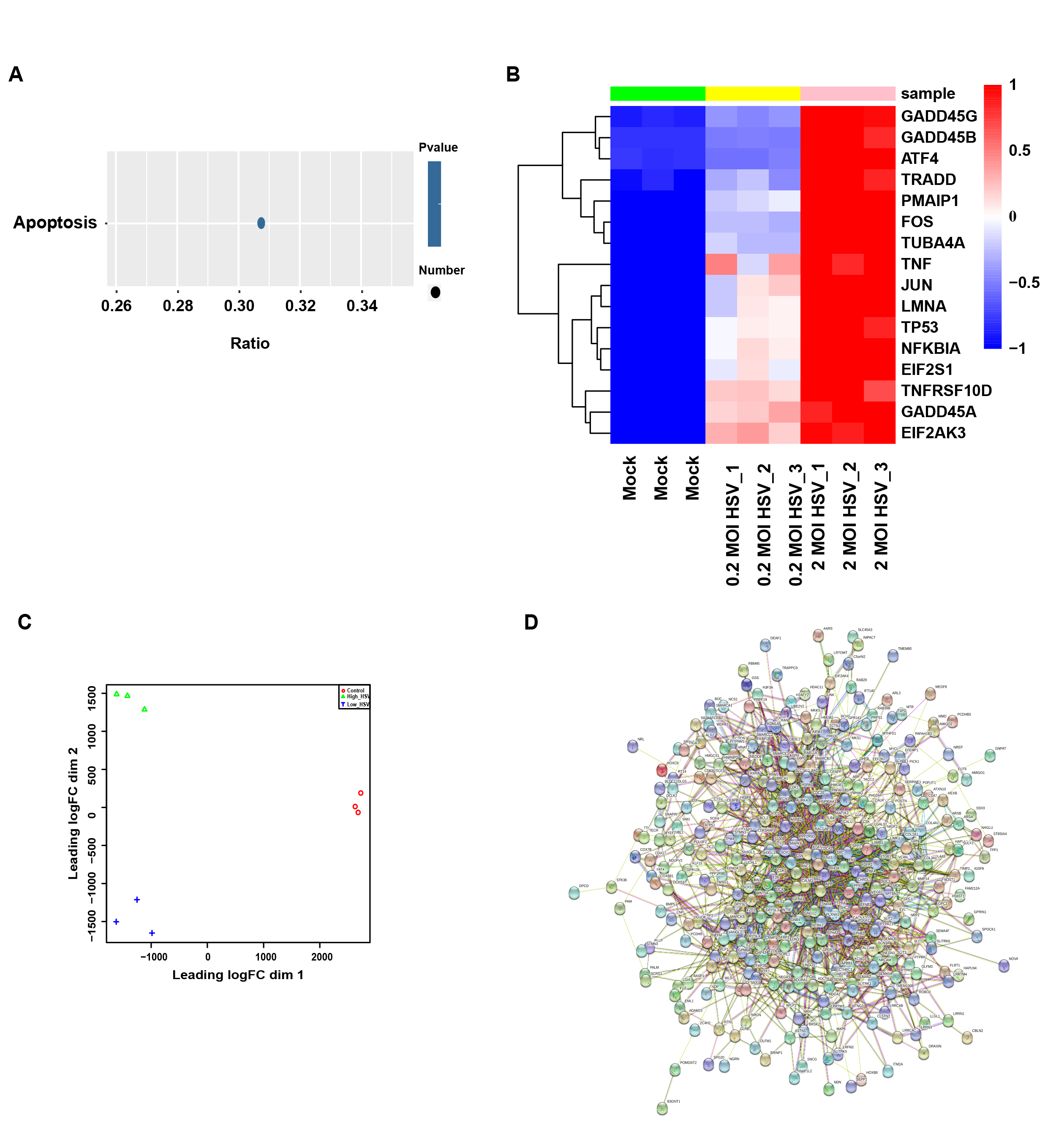

Supplement: S2 Fig — (A) Gene Ontology (GO) biological process groups enriched in genes down-regulated in NSCs infected with the HSV-1 (0.2 MOI), compared with the NSCs. (B) Heat map of apoptosis-related genes of RNA-seq dataset in HSV-1 infected NSCs. The apoptosis-related genes were obtained from http://www.genome.jp/kegg-bin/show_pathway?hsa04210/hsa:3708%09red/hsa:8503%09red/hsa:8793%09red/hsa:3725%09red/hsa:8797%09red/hsa:8794%09red/hsa:841%09red/hsa:843%09red/hsa:468%09red/hsa:330%09red/hsa:5414%09red/hsa:1521%09red/hsa:7124%09red/hsa:5604%09red/hsa:63970%09red/hsa:4616%09red/hsa:8743%09red/hsa:2353%09red/hsa:5170%09red/hsa:8739%09red/hsa:4914%09red/hsa:2081%09red/hsa:9451%09red/hsa:4792%09red/hsa:10018%09red/hsa:4790%09red/hsa:5366%09red/hsa:51807%09red/hsa:10912%09red/hsa:1647%09red/hsa:4000%09red/hsa:1514%09red/hsa:7277%09red/hsa:7157%09red/hsa:1147%09red/hsa:421.(C) PCA was performed based on transcriptomics data in the NSCs infected with or without HSV-1(0.2 MOI or 2 MOI) with a non-zero variance. (D) The key gene networks of the enriched pathways related to the neurogenesis and neuronal differentiation related to synapse organization, synapse assembly, neural tube development, nervous system development, axon guidance, cell morphogenesis involved in neuron differentiation, neuron projection guidance and neuron projection development based on the GO analysis. Search Tool for the Retrieval of Interacting Genes (STRING) was used to form key gene networks, the interactions with combined score ≥ 0.4 (medium confidence) were considered significant. (TIF) [file ppat.1008899.s002.tif]
